# Cervical Skip Metastases and Extensive Central Venous Thrombosis as the Initial Presentation of High‐Grade Prostate Adenocarcinoma With Discordant PI‐RADS 3 Findings: A Case Report and Diagnostic Challenge

**DOI:** 10.1002/ccr3.72920

**Published:** 2026-06-12

**Authors:** Yuchun Luo, Benhui Lai, Juncheng Huang

**Affiliations:** ^1^ Department of Urology Central Hospital of Guangdong Nongken Zhanjiang Guangdong Province China

**Keywords:** androgen deprivation therapy, apalutamide, cancer‐associated thrombosis, case report, cervical lymph node metastasis, PI‐RADS, prostate cancer, skip metastasis, superior vena cava thrombosis, venous thrombosis

## Abstract

Prostate cancer can initially present with cervical lymph node “skip” metastases and extensive central venous thrombosis, without pelvic or bone involvement. A PI‐RADS 3 lesion on mpMRI does not exclude high‐grade (Gleason 5 + 5 = 10) disease. Unexplained thrombosis in elderly men warrants prostate cancer investigation.

## 1 Introduction

Prostate cancer (PCa) is the most frequently diagnosed malignancy and a leading cause of cancer‐related mortality among men worldwide [1]. Its typical clinical trajectory involves local symptoms, such as lower urinary tract symptoms (LUTS), followed by metastasis that classically follows an orderly lymphatic drainage to the pelvic and retroperitoneal lymph nodes, with subsequent hematogenous spread to the axial skeleton, liver, and lungs [2]. The diagnosis is usually prompted by elevated serum prostate‐specific antigen (PSA) levels and is confirmed by histopathology, with multiparametric MRI (mpMRI) playing a central role in risk stratification and local staging [3].

However, the biological behavior of high‐grade PCa can deviate dramatically from these paradigms, presenting diagnostic challenges. Cervical lymph node involvement as the initial and sole presenting symptom of PCa is exceptionally rare, with only a handful of cases documented [4, 5]. Such presentations often cause diagnostic delay, as they mimic head and neck malignancies or lymphoma, particularly when typical genitourinary symptoms are absent.

We report a case of a 66‐year‐old man who presented with painful cervical lymphadenopathy as the first and only manifestation of an aggressive, high‐grade (Gleason score 5 + 5 = 10) prostate acinar adenocarcinoma. This case is remarkable for three converging factors. First, it represents a “skip” metastasis to the cervical chain in the complete absence of pelvic lymphadenopathy or bone involvement—a dissemination route that defies the classic stepwise progression model. Second, and most striking, is the profound discordance between the tumor's aggressive biology (PSA > 250 ng/mL, Gleason Grade Group 5) and its indolent mpMRI appearance, initially categorized as PI‐RADS 3 (equivocal), underscoring a critical limitation of imaging‐based risk assessment in high‐grade tumors [6]. Third, the disease was complicated by extensive thrombosis of the superior vena cava and bilateral internal jugular veins—a paraneoplastic phenomenon rarely reported as a presenting feature of PCa [7].

The confluence of these atypical features created a significant diagnostic conundrum. This case highlights the indispensable role of a broad immunohistochemical panel (including NKX3.1, PSA, and ERG) in tracing the origin of metastatic carcinoma of unknown primary [8], and demonstrates the remarkable efficacy of intensified androgen deprivation therapy (ADT) with apalutamide in achieving rapid biochemical and radiological remission even in this ultra‐high‐risk context.

By detailing this unusual presentation and its multidisciplinary management, this report aims to expand the understanding of prostate cancer dissemination, alert clinicians to the limitations of sole reliance on mpMRI in high‐grade disease, and reinforce the necessity of considering prostate origin in elderly men with unexplained cervical lymphadenopathy and thromboembolic events. We present the case in accordance with the CARE guideline.

## 2 Case Presentation

### 2.1 Medical History and Physical Examination

A 66‐year‐old man presented to our institution in July 2025 with a two‐week history of a painful, enlarging mass in the right cervical region. The mass, initially the size of a peanut, was associated with local erythema, warmth, and tenderness. He reported no lower urinary tract symptoms (LUTS), such as hesitancy, frequency, or nocturia, and no bone pain, hematuria, or constitutional symptoms like fever or night sweats. Empirical antibiotic therapy at a local hospital had been ineffective. His past medical, surgical, and family histories were unremarkable.

On physical examination, the patient was afebrile with stable vital signs (blood pressure 145/88 mmHg, pulse 100 bpm). A firm, tender, 2.5 × 2.0 cm right cervical lymph node was palpable, with limited mobility and slight adherence to surrounding tissues. The overlying skin was erythematous. The remainder of the examination, including digital rectal examination (DRE), was unremarkable; the prostate was noted to be enlarged but smooth and non‐nodular.

### 2.2 Initial Investigations and Diagnostic Workup

Initial laboratory tests revealed mild leukocytosis (white blood cell count 10.44 × 10^9^/L) with a normal neutrophil ratio. Contrast‐enhanced computed tomography (CT) of the chest and neck unexpectedly revealed extensive pathology: (i) multiple enlarged lymph nodes in the bilateral cervical chains (levels II–VII), mediastinum, and right hilum, the largest measuring 27 × 23 mm in left level IV (Figure 1A); (ii) filling defects consistent with thrombi in the superior vena cava (SVC), right common carotid vein, and bilateral internal jugular veins (Figure 1B); and (iii) multiple bilateral thyroid nodules and pulmonary nodules (Lung‐RADS 2 and 3). Abdominal ultrasound further identified a 15 × 8 mm hypoechoic nodule in the bladder trigone (Figure 2) and a heterogeneous prostate with calcifications. A vascular surgery consultation confirmed extensive central venous thrombosis, and elevated D‐dimer levels (4.83 mg/L) were documented. Due to local resource limitations, ^68^Ga‐PSMA PET/CT was not available; staging was completed with contrast‐enhanced CT and whole‐body bone scintigraphy.

**FIGURE 1:**
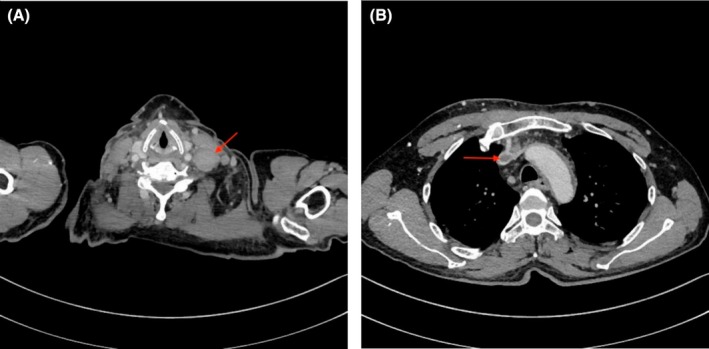
Initial contrast‐enhanced CT findings. (A) Axial CT image of the neck showing an enlarged left cervical lymph node at level IV, measuring 27 × 23 mm (arrow). (B) Coronal CT image demonstrating filling defects consistent with thrombi in the superior vena cava (SVC) (arrow).

**FIGURE 2:**
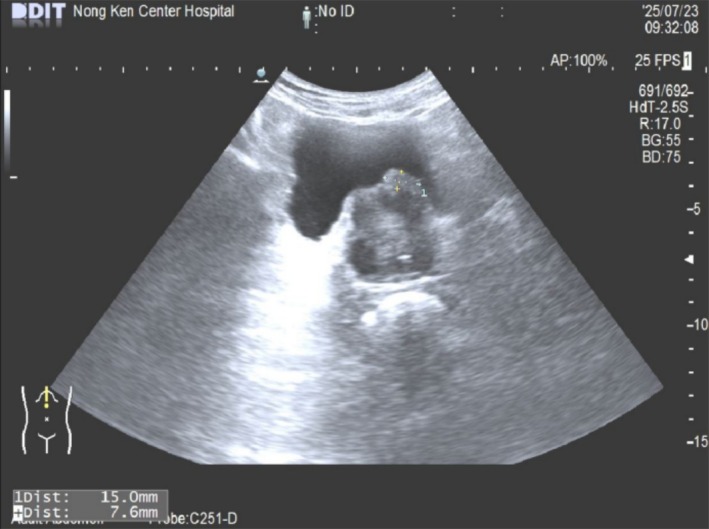
Transabdominal ultrasound showing a 15 × 8 mm hypoechoic nodule in the bladder trigone.

Given the suspicion of a malignant lymphoproliferative disorder vs. metastatic disease, the patient underwent an excisional biopsy of a left supraclavicular lymph node. Histopathology revealed metastatic carcinoma, and a focused immunohistochemical (IHC) panel was performed. The tumor cells were negative for lung (CK7, TTF‐1, Napsin A) and thyroid (Tg, Pax‐8) markers but showed strong positivity for p504s and NKX3.1, with a low Ki‐67 index (10%) (Figure 3). This IHC profile strongly suggested a prostatic origin, a hypothesis that was immediately pursued.

**FIGURE 3:**
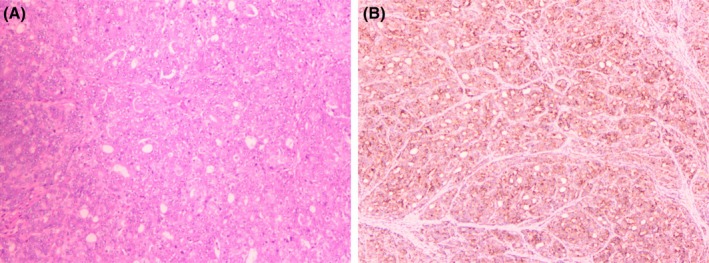
Left supraclavicular lymph node biopsy, 10 × objective. (A) H&E staining showing metastatic carcinoma. (B) Positive NKX3.1 IHC confirming prostatic origin. Scale bars = 100 μm.

Subsequent serum testing revealed a markedly elevated total PSA of 254.7 ng/mL and a free PSA of 16.68 ng/mL (free/total ratio 0.07). Multiparametric MRI (mpMRI) of the prostate was performed for local staging. It demonstrated an enlarged prostate (49 × 40 × 57 mm) with a poorly defined area in the anterior transition zone showing mild diffusion restriction (b = 1,000) and an equivocal finding on dynamic contrast enhancement, resulting in an overall PI‐RADS v2.1 assessment category of 3 (Figure 4). Crucially, there was no evidence of pelvic lymphadenopathy, seminal vesicle invasion, or bone destruction on pelvic MRI or whole‐body bone scintigraphy. Brain MRI revealed only chronic microvascular changes.

**FIGURE 4:**
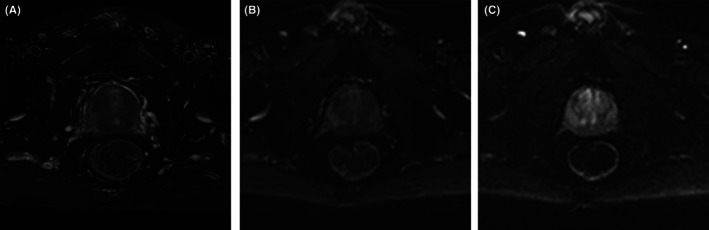
Multiparametric MRI of the prostate. Axial T2‐weighted image (A), diffusion‐weighted image (b = 1,000) (B), and ADC map (C) show an ill‐defined area in the anterior transition zone with mild restricted diffusion, classified as PI‐RADS 3.

### 2.3 Definitive Diagnosis

To obtain a definitive tissue diagnosis from the primary site, the patient underwent transurethral resection of the prostate (TURP) and resection of the bladder nodule. Histopathology of the prostate chips confirmed a bilateral, high‐grade prostate acinar adenocarcinoma with a Gleason score of 5 + 5 = 10 (Grade Group 5), characterized by solid sheets and nests of poorly differentiated tumor cells (Figure 5A). The bladder lesion was confirmed as metastatic adenocarcinoma of prostatic origin. A comprehensive IHC panel on the prostate tissue was strongly positive for PSA, NKX3.1, ERG, and P504S, with retained p53 expression and a very low Ki‐67 index (2%), confirming the diagnosis and lineage (Figure 5B). The final clinicopathological stage was cT4N1Mx, reflecting local bladder invasion and distant nodal metastasis.

**FIGURE 5:**
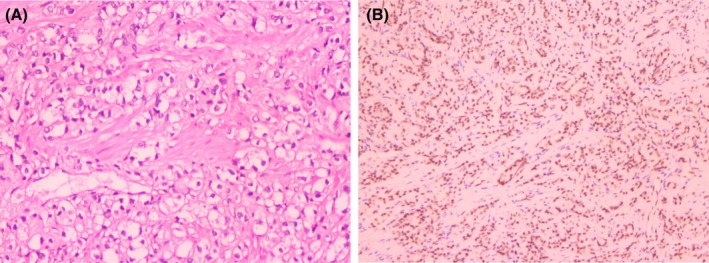
TURP histopathology, 20 × objective. (A) H&E staining showing Gleason score 5 + 5 = 10 prostate adenocarcinoma. (B) IHC confirming prostatic origin with diffuse NKX3.1 positivity. Scale bars = 50 μm.

### 2.4 Therapeutic Intervention and Follow‐Up

The patient was started on combination therapy with androgen deprivation therapy (ADT) using the GnRH antagonist degarelix (loading dose 240 mg, then 80 mg monthly subcutaneously) and the second‐generation androgen receptor inhibitor apalutamide (240 mg orally daily). Anticoagulation with rivaroxaban (10 mg daily) was initiated for the central venous thrombi.

The treatment response was rapid and profound. PSA levels declined precipitously from 254.7 ng/mL at baseline to 35.8 ng/mL at one month, 19.9 ng/mL at two months, and 5.07 ng/mL at six months, with sustained testosterone suppression (< 0.2 μg/L) (Figure 6). Follow‐up mpMRI at three and six months demonstrated a marked reduction in the size of the primary prostate lesion, which now showed no residual restricted diffusion, and a significant regression of the left cervical lymphadenopathy (from 23 × 16 mm to 21 × 12 mm) (Figure 7). The SVC and jugular vein thrombi completely resolved on follow‐up imaging by three months, allowing for the safe discontinuation of rivaroxaban. The patient tolerated the regimen well, with no significant adverse events or laboratory abnormalities.

**FIGURE 6:**
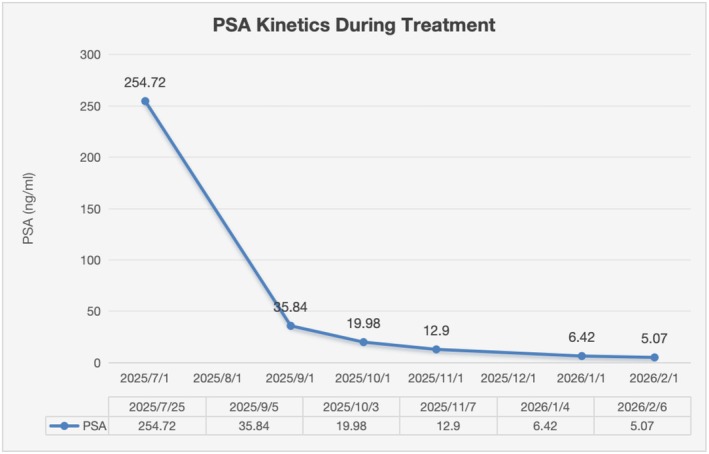
PSA kinetics during treatment. Serum PSA levels declined rapidly from 254.7 ng/mL at baseline to 5.07 ng/mL at six months following initiation of degarelix and apalutamide, with sustained testosterone suppression.

**FIGURE 7:**
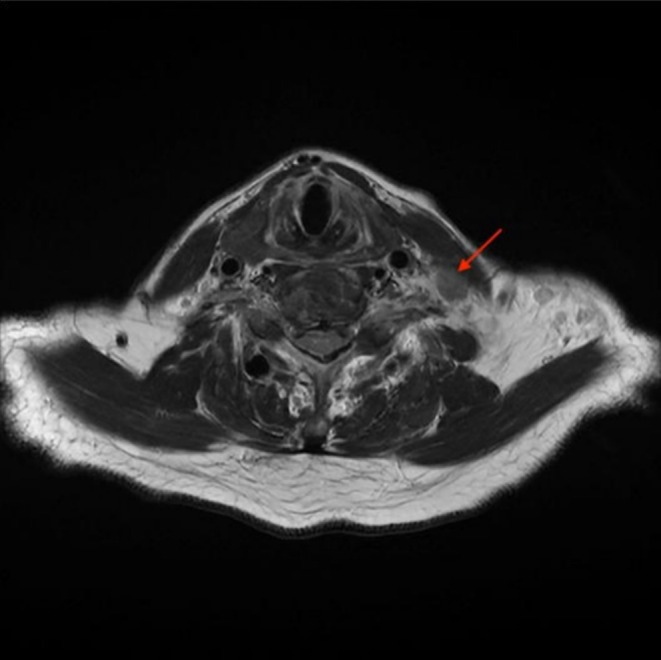
Six‐month follow‐up MRI showing regression of left cervical lymph node metastases (arrow) following treatment.

In summary, this case presents a 66‐year‐old man with a diagnosis of high‐grade (Gleason 5 + 5 = 10) prostate acinar adenocarcinoma, uniquely characterized by symptomatic cervical lymph node skip metastases and extensive central venous thrombosis as the initial presentation, in the absence of typical pelvic or bone involvement. The case highlights a profound discordance between aggressive tumor biology and equivocal mpMRI findings (PI‐RADS 3).

## 3 Discussion

We report a highly unusual case of high‐grade prostate acinar adenocarcinoma (Gleason score 5 + 5 = 10) that presented exclusively with painful cervical lymphadenopathy and extensive central venous thrombosis, in the complete absence of typical lower urinary tract symptoms, pelvic lymph node involvement, or bone metastases. This case is instructive for several reasons: It represents a rare “skip” metastatic pattern, highlights a profound discordance between aggressive tumor biology and equivocal mpMRI findings (PI‐RADS 3), and demonstrates a successful multidisciplinary approach to diagnosis and management. We discuss the implications of these findings for clinical practice and our understanding of prostate cancer biology.

### 3.1 A Paradigm‐Defying Metastatic Pattern: Skip Metastasis to Cervical Nodes

The classical model of prostate cancer dissemination posits an orderly progression from the primary tumor to regional pelvic lymph nodes, followed by retroperitoneal nodes, and ultimately hematogenous spread to the axial skeleton and viscera [9]. Our case fundamentally challenges this paradigm. The presence of bulky cervical lymph node metastases in the absence of any detectable pelvic or retroperitoneal nodal involvement represents a true “skip metastasis.” While extremely rare, this phenomenon has been sporadically reported and may be more common in high‐grade, aggressive tumors [5]. The underlying mechanisms remain speculative but may involve: (i) hematogenous dissemination through the vertebral venous plexus (Batson's plexus), which bypasses the caval system and provides a direct conduit to the cervical spine and nodes [10]; (ii) tumor cell trafficking via the thoracic duct with retrograde flow into cervical lymphatic trunks, as has been described in the broader context of lymph node metastasis in prostate cancer [11]; or (iii) unique molecular signatures that confer a tropism for lymphatic endothelium in the cervical region [12]. Regardless of the precise mechanism, this case serves as a critical reminder that prostate cancer must remain in the differential diagnosis for cervical lymphadenopathy of unknown origin in elderly men, even when typical pelvic findings are absent.

### 3.2 The Great Masquerader: Aggressive Biology Behind Benign Imaging

Perhaps the most clinically significant aspect of this case is the striking discordance between the tumor's aggressive biological behavior—evidenced by a PSA > 250 ng/mL, Gleason Grade Group 5 histology, and distant metastatic spread—and its remarkably indolent appearance on mpMRI, which was categorized as PI‐RADS 3 (equivocal). Multiparametric MRI has become an indispensable tool in prostate cancer diagnosis and risk stratification, with PI‐RADS scoring guiding biopsy decisions and treatment planning [13, 14]. However, its limitations in detecting high‐grade tumors, particularly those with infiltrative, non‐nodular growth patterns or those located in the anterior transition zone, are increasingly recognized [15]. Some poorly differentiated tumors may lack the discrete nodular architecture and neoangiogenic vascularity that underlie the typical MRI findings of restricted diffusion and early enhancement [16].

Several factors may converge to produce such a profound imaging–biology discordance. First, anterior prostate cancers—such as the one in our patient—frequently present with higher PSA density yet can remain occult on mpMRI due to poor lesion conspicuity, background stromal hyperplasia, and limited neoangiogenesis. Emerging data suggest that up to 16%–25% of PI‐RADS 3 lesions harbor clinically significant prostate cancer (csPCa), and the risk is substantially higher when PSA density exceeds 0.15 ng/mL/mL or serum PSA is markedly elevated [17]. In such settings, a PI‐RADS 3 score should not reassure the clinician; rather, it should mandate systematic and possibly targeted biopsy, especially when accompanied by suspicious findings such as proven metastases or a PSA level above 100 ng/mL. Independent review of PI‐RADS 3 examinations at high‐volume reference centers has been shown to upgrade a substantial proportion of cases and improve diagnostic accuracy [17].

Second, the histological pattern itself contributes to the MRI invisibility. Gleason grade 5 tumors may form solid sheets, cribriform structures, or single infiltrative cells that do not create the dense cellular nodules required to generate marked diffusion restriction. Invasive cribriform and intraductal carcinoma patterns, which frequently coexist with high‐grade acinar adenocarcinoma and are recognized for their aggressive behavior, are also easily underestimated by conventional PI‐RADS assessment, and their presence portends a significantly worse disease‐specific survival [18]. Although our case was dominated by solid sheets without definitive cribriform glands, the confluent architecture fits the grade 5 designation and likely shares the same challenges in MRI conspicuity. This underscores the need for a high index of suspicion and, when serum markers are alarmingly elevated, prostate biopsy regardless of imaging findings.

Third, underlying genomic alterations may uncouple cell density from imaging signals. Emerging radiogenomic frameworks that integrate imaging features with gene expression data, as shown in prostate cancer [19] and renal cell carcinoma [20], could eventually identify a “high‐risk PI‐RADS 3” molecular subtype, guiding personalized diagnostic pathways.

### 3.3 Thrombosis as a Harbinger: Expanding the Paraneoplastic Spectrum

The extensive thrombus formation involving the superior vena cava and bilateral internal jugular veins as a presenting feature adds another layer of complexity to this case. Cancer‐associated thrombosis (CAT) is a well‐recognized complication of malignancy, driven by a multifactorial hypercoagulable state involving tumor‐derived procoagulant factors, tissue factor expression, endothelial injury, and stasis [21]. While adenocarcinoma of the pancreas, lung, and gastrointestinal tract are most commonly associated with CAT, prostate cancer is an under‐recognized but important cause [22]. Trousseau's syndrome—migratory thrombophlebitis—has been described in prostate cancer, but extensive central venous thrombosis at initial presentation is exceptionally rare [23]. The location of the thrombi in our patient, contiguous with the bulky cervical nodal metastases, suggests a possible local vascular compressive or infiltrative component in addition to the systemic hypercoagulable state. This case highlights that unexplained thrombosis in atypical locations, particularly in an older man, should prompt a comprehensive investigation for an occult malignancy, including prostate cancer. Furthermore, it demonstrates that targeted anticoagulation can be safely and effectively integrated with systemic cancer therapy, leading to complete thrombus resolution, as shown with direct oral anticoagulants in cancer‐associated thrombosis [24].

### 3.4 Therapeutic Implications: Efficacy of Intensified Hormonal Therapy in Ultra‐High‐Risk Disease

The rapid and deep biochemical and radiographic response to combined ADT and apalutamide observed in this patient is noteworthy. The patient achieved a > 95% reduction in PSA within two months and sustained suppression at six months, accompanied by significant regression of both the primary tumor and cervical metastases. This aligns with the results of landmark trials such as TITAN and SPARTAN, which demonstrated the superiority of adding apalutamide or enzalutamide to ADT in metastatic hormone‐sensitive prostate cancer (mHSPC) [25, 26]. Our case extends these findings by illustrating the remarkable efficacy of this regimen even in the context of an ultra‐high‐risk, non‐classical metastatic presentation complicated by thrombosis. It supports the use of intensified combination therapy as first‐line treatment for such aggressive cases and suggests that the biology of the tumor, rather than the specific anatomic pattern of spread, should be the primary driver of treatment intensity.

### 3.5 Strengths, Limitations, and Future Directions

This report has several strengths. The diagnosis is unequivocally established by comprehensive histopathology and immunohistochemistry from both the metastatic and primary sites, with longitudinal PSA and mpMRI follow‐up providing robust response evidence. However, limitations must be acknowledged. This is a single case with short follow‐up; long‐term durability and castration resistance remain unknown. We lacked comprehensive genomic profiling (TP53, RB1, PTEN, BRCA1/2) [27] and PSMA PET/CT staging, now recommended for high‐risk disease [28, 29]; PET would have better delineated disease extent. The precise skip‐metastasis mechanism remains speculative without functional studies. Future work should incorporate genetic testing for homologous recombination repair mutations to guide potential PARP inhibitor therapy [30], and systematic multi‐omics data collection will be essential to model imaging‐biology discordance.

### 3.6 Take‐Home Messages for Clinicians

Several specific, case‐driven lessons emerge from this report:

(1) In men ≥ 60 years with unexplained cervical lymphadenopathy or atypical central venous thrombosis, PSA measurement is mandatory regardless of urinary symptoms or DRE findings.

(2) A PI‐RADS 3 lesion does not exclude high‐grade cancer; when PSA exceeds 100 ng/mL or PSA density is elevated, systematic biopsy and expert MRI re‐review should be pursued.

(3) Metastatic carcinoma of unknown primary in male patients demands a prostate‐oriented IHC panel (NKX3.1, PSA, PSMA, ERG). Unexplained extensive venous thrombosis should similarly trigger prostate cancer workup, and anticoagulation can safely be combined with ADT.

(4) Early BRCA1/2 testing is advised for high‐volume metastatic disease to inform future PARP inhibitor use. Even ultra‐high‐risk, atypically metastatic prostate cancer can show dramatic responses to apalutamide‐based intensified therapy, emphasizing that tumor biology–not anatomy–should dictate treatment intensity.

## 4 Conclusion

In conclusion, this case provides a unique window into the heterogeneous and sometimes unpredictable behavior of high‐grade prostate adenocarcinoma. It expands the phenotypic spectrum of the disease to include cervical skip metastases and extensive central venous thrombosis as initial presenting features, while simultaneously exposing a critical blind spot in MRI‐based risk stratification. By documenting this case, we hope to raise clinical awareness, reduce diagnostic delays, and encourage further research into the molecular underpinnings of aberrant metastatic spread and imaging–pathology discordance in prostate cancer. Future studies that integrate molecular profiling with advanced imaging are needed to refine diagnostic algorithms and lead to more personalized therapeutic strategies for patients with this challenging disease.

## Author Contributions

**Yuchun Luo:** conceptualization, methodology, data curation, investigation, validation, formal analysis, funding acquisition, writing – original draft. **Benhui Lai:** data curation, investigation. **Juncheng Huang:** writing – review and editing, project administration, supervision.

## Funding

This work was supported by the Guangdong Medical Research Foundation (B2025453).

## Ethics Statement

This case report was conducted in accordance with the ethical standards of the institutional research committee and with the Helsinki Declaration. The study was approved by the Institutional Review Board of the Central Hospital of Guangdong Nongken. Written informed consent was obtained from the patient for participation.

## Consent

Written informed consent for publication of this case report and any accompanying images was obtained from the patient. A copy of the written consent is available for review by the Editor‐in‐Chief of this journal upon request.

## Conflicts of Interest

The authors declare no conflicts of interest.

## Data Availability

The data that support the findings of this study are available on request from the corresponding author. The data are not publicly available due to privacy or ethical restrictions.
